# Supplementary material for: Evaluation of clinical outcomes of patients with mild symptoms of coronavirus disease 2019 (COVID-19) discharged from the emergency department
Source: PLoS One. 2021 Oct 21;16(10):e0258697. doi: 10.1371/journal.pone.0258697 (PMC8530279; doi:10.1371/journal.pone.0258697)
Supplement: S1 File — (DOC) [file pone.0258697.s001.doc]

S1 File: Original version (Persian) of relevant parts of the questionnaires used in the study.

چک لیست

نام : نام خانوادگی:

سن: جنس: شماره تماس در دسترس:

وضعیت تاهل:

نوع اشتغال:

تعدا افراد خانواده:

علایم حیاتی:

فشار خون: تعداد ضربان قلب: تعداد تنفس:

: GCS : O2SAT دمای بدن:

PMH

سابقه دیابت: سابقه فشار خون بالا: سایر موارد:.................

شکایت اولیه:

زمان شروع شکایات:

رژیم دارویی هنگام ترخیص از اورژانس.

پیامد بیماری در روز هفتم: حال عمومی........ نوع درمان......نوع مراجعه:.......

پیامد بیماری در روز بیست و یکم: حال عمومی........ نوع درمان......نوع مراجعه:.......

پیامد بیماری در روز سی ام: حال عمومی........ نوع درمان......نوع مراجعه:.......
