# Supplementary material for: Evaluation of clinical outcomes of patients with mild symptoms of coronavirus disease 2019 (COVID-19) discharged from the emergency department
Source: PLoS One. 2021 Oct 21;16(10):e0258697. doi: 10.1371/journal.pone.0258697 (PMC8530279; doi:10.1371/journal.pone.0258697)
Supplement: S2 File — English translation and so original version (Persian) of relevant parts of the questionnaires used in the study. (DOC) [file pone.0258697.s002.doc]

[**S1 File.**](https://journals.plos.org/plosone/article/file?type=supplementary&id=info:doi/10.1371/journal.pone.0256142.s002)**Questionnaire.**

English translation of relevant parts of the questionnaires used in the study.

**Check list**

- **Name and family name**…………….…
- **Age: ……………**
- **Gender…………**
- **Contact number available………………**
- **Marital status: …………….**
- **Type of employment…………………**
- **Number of family members**...............
- **Vital signs……………………..**

Blood pressure… Heart rate: …….. Respiration rate… GCS……. O2SAT………… Body temperature: ………

- **PMH**

History of diabetes…………..

History of high blood pressure………….…

Other cases: .................

- **Initial complaint……………**
- **Complaints start time: ………….**
- **Medication regimen during emergency discharge…………………………**
- **Outcome of the disease on 7th day**:

General condition........

Type of treatment......

Type of referral: .......

- **Outcome of the disease on the 21th day:**

General condition........

Type of treatment......

Type of referral: .......

- **Outcome of the disease on the 30th day:**

General condition........

Type of treatment......

Type of referral: .......
