# Supplementary material for: Evaluation of clinical outcomes of patients with mild symptoms of coronavirus disease 2019 (COVID-19) discharged from the emergency department
Source: PLoS One. 2021 Oct 21;16(10):e0258697. doi: 10.1371/journal.pone.0258697 (PMC8530279; doi:10.1371/journal.pone.0258697)
Supplement: Attachment — Submitted filename: Dear Respected Editor and reviewers.doc [file pone.0258697.s003.doc]

**Dear Respected Editor and reviewers**

**Many thanks for your comprehensive comments regarding our article. Herewith, we revised our article based on your advice in the best manner as follows:**

**Reviewer #1:**

**Dear Reviewer 1
I appreciate you for reviewing our manuscript so meticulously and mentioning very important comments. Below you could find our reply to your comments.**

**Q1: The authors do not discuss the specifics of the hospital involved in the study.**

¬ Reply:  Based on your comment, we explained the specifics of the hospitals involved in the study in the Method section.

**Q2: Please mention the weak and strong points of your study**

¬ Reply:  Based on your comment, we explained the weak and strength of study in end of discussion section.

**Reviewer #2:**

**Dear Reviewer 2
I appreciate you for reviewing our manuscript so meticulously and mentioning very important comments. Below you could find our reply to your comments.**

**Q 1-Is there any information regarding the severity scores like APACHE or SAPS in the study population?**

¬ Reply:  Thank you so much for reviewing our manuscript meticulously. Since our included patients were outpatient’s patients we couldn’t collected data for calculating the APACHE or SAPS due to cost problem. Furthermore, based on the literature and experts’ opinion the mentioned predictor scores is not suitable for COVID-19 patients.

**Q2- There should be a table showing demographic characteristics of the patients in the study.**

¬ Reply:  I really appreciate you for your comprehensive comments. We provided 3 tables (1, 2 and 5) regarding your nice advice.

**Q3-is there any information about the duration of ED stay, method of ventilation, prescribed drugs, antiviral drugs...?**

¬ Reply:  We studied discharged outpatient patients from ED no need to ventilation or ED stay. The prescribed drug is mentioned in Table 4.

**Q4-What was the criteria used for mild category of COVID-19 in this study?**

¬ Reply:  We meant those patients in good general condition, oxygen level (SpO2) over 90%, non-intubated, and having mild fever and also who tested positive for SARS-CoV-2 by reverse transcription polymerase chain reaction (RT-PCR) assay of nasopharyngeal swabs.

**Q5-What was the criteria for discharge of patients from ED?**

¬ Reply:  We discharged patients in good general condition, oxygen level (SpO2) over 90%, non-intubated, and having mild fever and also who tested positive for SARS-CoV-2 by reverse transcription polymerase chain reaction (RT-PCR) assay of nasopharyngeal swabs.

**6-it is better to show the any information about ED revisits, inpatient admissions, ICU admissions after discharge from hospital.**

¬ Reply:  Thank you for your valuable comment. The required data has been show in the Table 6 and figure 1

**7-How did the authors delete the effect of confounding factors from the study outcome?**

Reply- :  We meant those patients in good general condition, oxygen level (SpO2) over 90%, non-intubated, and having mild fever and also who tested positive for SARS-CoV-2 by reverse transcription polymerase chain reaction (RT-PCR) assay of nasopharyngeal swabs.

**8-Discussion should be improved and revised based on the study results and comparison with previous ones.**

Reply- Many thanks for your attention. We did comprehensive review and improved the discussion section by the similar studies.

**Q9-What is the new finding of this study compared to previous ones the this field?**

Reply- We discussed the mortality, Household Transmission of COVID-19 and Hygiene Compliance Rate and so revisit of the Mild covid-19 outpatients discharged from ED.

**10-Is there any data regarding economic situation of patients,as previous studies showed an important economic consequence of the disease at an early post-hospital phase.**

Reply- In Table 1 the patients’ job is explained.

**11-What was the limitation of the study?**

Reply-  Based on your comment, we explained the weak and strength of study in end of discussion section.
